# Motor Task Variation Induces Structural Learning

**DOI:** 10.1016/j.cub.2009.01.036

**Published:** 2009-02-24

**Authors:** Daniel A. Braun, Ad Aertsen, Daniel M. Wolpert, Carsten Mehring

**Affiliations:** 1 Computational and Biological Learning Lab, Department of Engineering, University of Cambridge, Cambridge CB2 1PZ, UK; 2 Bernstein Center for Computational Neuroscience, Albert Ludwigs University, D-79104 Freiburg, Germany; 3 Institute of Biology I, Albert Ludwigs University, D-79104 Freiburg, Germany; 4 Institute of Biology III, Albert Ludwigs University, D-79104 Freiburg, Germany

**Keywords:** SYSNEURO

## Abstract

When we have learned a motor skill, such as cycling or ice-skating, we can rapidly generalize to novel tasks, such as motorcycling or rollerblading [1–8]. Such facilitation of learning could arise through two distinct mechanisms by which the motor system might adjust its control parameters. First, fast learning could simply be a consequence of the proximity of the original and final settings of the control parameters. Second, by structural learning [9–14], the motor system could constrain the parameter adjustments to conform to the control parameters' covariance structure. Thus, facilitation of learning would rely on the novel task parameters' lying on the structure of a lower-dimensional subspace that can be explored more efficiently. To test between these two hypotheses, we exposed subjects to randomly varying visuomotor tasks of fixed structure. Although such randomly varying tasks are thought to prevent learning, we show that when subsequently presented with novel tasks, subjects exhibit three key features of structural learning: facilitated learning of tasks with the same structure, strong reduction in interference normally observed when switching between tasks that require opposite control strategies, and preferential exploration along the learned structure. These results suggest that skill generalization relies on task variation and structural learning.

## Results and Discussion

**Figure 1:**
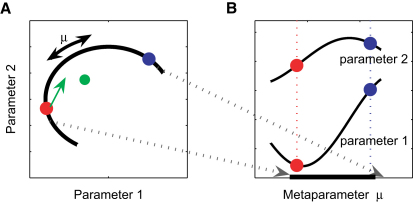
Schematic Diagram of Structural Learning (A) The task space is defined by two parameters, but for the given task, only certain parameter combinations occur (black line). This relationship is indicated by the curved structure, which can be parameterized by a one-dimensional metaparameter μ. However, a parametric learner that is ignorant of the structure has to explore the full two-dimensional space when readjusting the parameter settings. (B) A structural learner, in contrast, takes the relationship between the parameters into account. By adjusting only the metaparameter μ, the learning problem is effectively one-dimensional.

Motor learning is often regarded as a process of learning a new mapping from sensory inputs to motor outputs [1–8]. Such mappings can be represented, for example, by simple feed-forward neural networks, and learning can be achieved by adjusting synaptic weight parameters in these networks (e.g., radial basis function networks) [2, 6, 15, 16]. The solution of a control problem can then be represented as a setting of these parameters—Figure 1A shows a schematic of a simple two-parameter system. For example, the red setting could be the solution for riding a racing bike, and the blue setting could be the solution for riding a mountain bike. Learning the control process of a mountain bike when having already learned that of a racing bike corresponds then to changing the parameter setting from red to blue. Thus, learning becomes a search through parameter space. How could we speed up this learning process? Clearly, if the old and new parameter settings are close to each other in parameter space, then learning can be fast. However, there is another possible way to speed up learning. If we have ridden many different types of bicycles, we might have extracted general rules for how the control parameters covary for different bicycles. That is, the set of bicycles may not span the entire parameter space, but lie on a low-dimensional subspace (e.g., the thick black line in Figure 1A), termed a structure. Learning such a structure would be beneficial in guiding exploration of the parameter space for a new bicycle. It would allow us to introduce a new metaparameter (with setting μ) that adjusts the control parameters to move along the lower-dimensional structure in parameter space (Figure 1B). Therefore, when we are presented with a new task on the same structure, the search is restricted to a subspace of the full parameter space (e.g., the control subspace for the class of all bikes), thereby speeding up learning.

Such structural learning [9–14] would have three clear benefits. First, by reducing the search space from a high-dimensional space to a low-dimensional space, the efficiency of any learning algorithm will be dramatically improved [17]. Therefore, we expect structure-specific facilitation for tasks that conform to a learned structure; that is, learning should be faster compared to that for tasks that lie off the structure (Figure 1A, green setting). Second, when two tasks that require opposite control strategies, such as opposing visuomotor rotations, are learned consecutively, the first task makes it more difficult to learn the second task (anterograde interference), and the second task wipes out memory of the first (retrograde interference) [18, 19]. However, if two opposing perturbations (e.g., ±60° visuomotor rotations) could be learned as part of the same structure (e.g., rotation structure), then we would expect a low-dimensional, high-speed pathway between the parameter settings for the two opposing perturbations. This should be reflected in reduced anterograde and retrograde interference between opposing tasks (structure-specific interference reduction). Third, when moving between tasks belonging to the same structure, the controller should preferentially explore along the structure (the thick black line in Figure 1A) and reduce deviations from the structure. Moreover, for a task that lies off the current structure (green setting), the initial exploration (green arrow when starting from red disk) should still lie preferentially along the structure (structure-specific exploration).

To investigate structural learning, we devised a series of experiments in which subjects were exposed to visuomotor transformations in different virtual reality environments. Numerous studies have shown that subjects can rapidly adapt to a fixed visuomotor transformation, such as a rotation induced by prism glasses [7]. Here, however, we varied the parameters of such visuomotor transformations randomly over trials, while leaving the structure of the transformation the same. For example, we randomly varied the rotation angle (the parameter) of a visuomotor rotation (the structure) between the actual and the visually perceived location of the subject's hand. In such randomly varying situations, previous studies suggest that subjects cannot represent these multiple transformations and only learn the average [20–25]. Here, we introduce probe trials to investigate whether, despite the apparent lack of learning, subjects show evidence of structural learning.

### Learning of Visuomotor Rotations

**Figure 2:**
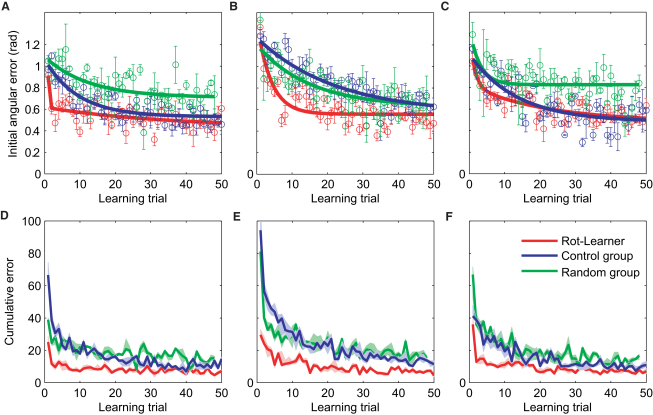
Structural Learning of Visuomotor Rotations (A) Learning curves for a block of +60° rotation trials performed by a group that had experienced random rotations before (Rot-learner, red), a control group that had only experienced movements with veridical feedback (blue), and a group that experienced random linear transforms (green). The rotation group shows strong facilitation. The initial angular error over all subjects is shown with double-exponential fits. (B) Learning curves for a subsequent block of −60° rotation trials performed by the same groups. The interference effect that can be seen in the control group is strongly reduced in the rotation group. (C) Learning curves for a subsequent block of +60° rotation trials performed by the same groups. Again, the random rotation group shows a performance advantage in the first ten trials. (D–F) The same effects are much more pronounced for the cumulative error computed over the entire trajectory. Facilitation (D), interference reduction (E), and facilitation of relearning (F) are significant. The median error over all subjects and the pertinent interquartile confidence interval are shown.

To test for structure-specific facilitation, we exposed a group of subjects to an extended period (800 trials) of visuomotor rotations during planar reaching, with the rotation angle varying randomly. The rotation angle changed every eight trials (drawn uniformly from between −90° and +90°), and within these trials, each of the eight possible targets was presented once in a pseudorandom order (see Supplemental Experimental Procedures, available online, for details). A control group performed similarly but without a visuomotor transformation. Both groups were then exposed to a fixed visuomotor rotation of +60°. We examined learning of the +60° rotation on the basis of two error measures. First, we analyzed the initial angular error (200 ms after movement onset) to assess learning of the feed-forward control command (Figures 2A–2C). Second, we calculated a cumulative-error measure over the entire trajectory to assess the joint learning effect of feed-forward and feedback control (Figures 2D–2F). Compared to the controls, the random rotation group showed significant (p < 0.01, Wilcoxon rank-sum test on mean error over the first ten trials) facilitation of feed-forward learning in the +60° rotation block (Figure 2A, red versus blue). Moreover, the random rotation group generally moved faster, and movement durations were accordingly reduced in this group (p < 0.001, Wilcoxon rank-sum test on mean duration). These faster movements were also more accurate overall, as can be seen in the cumulative-error measure computed for the entire movement trajectories (Figure 2D, red versus blue, p < 0.001, Wilcoxon rank-sum test on mean error over the first ten trials). Thus, random rotation experience not only led to facilitated feed-forward learning of rotations, but also to improved feedback control.

To rule out the possibility that this facilitation was simply due to exposure to a similar rotation angle in the directly preceding rotation blocks of the random rotation group, we correlated the movement error of the first trial in the +60° rotation block with the rotation angle of the last two random blocks preceding the +60° block. We found no significant correlation between initial movement error in the +60° rotation block and previous rotation angles (r^2^ < 0.07 for the preceding random block and r^2^ < 0.001 for the penultimate random block). Moreover, the mean rotation angle before the +60° rotation block was −12° (for the penultimate block it was −6°), which means that even if there had been a strong correlation, it could have only been to the disadvantage of the random rotation group. Therefore, the strong facilitation effect cannot be explained by a simple memorization of the directly preceding rotation trials. To further test that the observed facilitation is also not a net effect of memorizing all previous rotation experiences close to +60° (see below for the case of −60° rotations), we introduced another group that experienced the same amount of ±60° rotations in the exposure phase as the random rotation group. Whenever the randomly chosen angle fell in the range +50° and +70° or −50° and −70°, the subjects experienced a +60° or −60° rotation, respectively; otherwise, they experienced a random linear transformation composed of a rotation, a shearing, and a scaling (Supplemental Experimental Procedures for details). Thus, this random linear group experienced the same amount of memorable ±60° rotations; however, this was not in the context of a rotation structure, but of a much less constrained linear transform structure. We found that the random linear group performed worse than the random rotation group in the +60° rotation block (p < 0.01), both in terms of feed-forward learning and feedback control (Figures 2A and 2D, green versus red). In fact, the cumulative error of the random linear group was more similar to that of the naive control group (Figure 2D, green versus blue), which suggests that the subjects did not benefit from previous ±60° rotation trials that were embedded in the random linear structure. Their feed-forward learning was even slower than the naive controls' errors (Figure 2A, green versus blue), suggesting that the random linear group had mainly learned to rely on feedback control.

After the +60° rotation block, all three groups were exposed to the opposite visuomotor rotation of −60°. The naive control group showed a significant decrement in feed-forward learning (Figure 2B, blue) compared to the learning of the +60° (Figure 2A, blue), consistent with many studies that have shown anterograde interference between opposing visuomotor rotations [18, 19]. We observed the same interference effect for the random linear group (Figure 2B, green). Although the random rotation group had learned the +60° rotation better than the other groups, they showed a significant reduction (p < 0.01) in interference (Figure 2B, red). When comparing the cumulative error that also takes feedback control into account (Figure 2E, red), the interference reduction of the random rotation group was even more pronounced (p < 0.001) compared to that of the other two groups (Figure 2E, blue and green).

Finally, all three groups were exposed to the original +60° rotation (Figures 2C and 2F). Again, we see a trend in the initial learning of the second +60° rotation block (Figure 2C, red versus blue) showing that feed-forward learning in the random rotation group was facilitated compared to that in the naive group (p < 0.02, Wilcoxon rank-sum test on mean error over the first ten trials). This difference is more pronounced in the cumulative error (p < 0.01, Wilcoxon rank-sum test on mean error over the first ten trials; Figure 2F) assessing feed-forward and feedback control. The control group and the linear random group showed a decrement in performance compared to that in the last few trials in the original exposure to the +60°. This retrograde interference was markedly reduced for the random rotation group (Figure 2F, red). Taken together, the results of this experiment suggest that the experience of a single structure whose parameters vary continuously over a range leads to both structure-specific facilitation and structure-specific interference reduction. Furthermore, this structure-specific performance enhancement seems to have a feedback and a feed-forward component.

### Learning of Shearings versus Rotations

**Figure 3:**
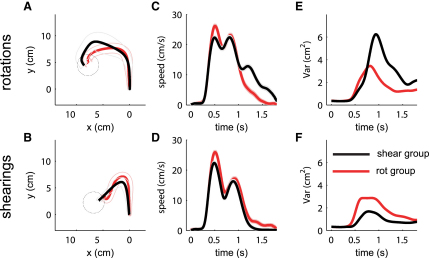
Structural Learning of Rotations versus Shearings (A) Mean trajectories over all subjects in 60° rotation probe trials performed by a group that experienced random rotations before (red) and another group that experienced random shearings before (black). The two groups react differently to the same perturbation. The trajectories to the different targets have all been rotated so that the cursor target is vertically above the starting location. (B) Mean trajectories in 1.5 shearing probe trials performed by the same groups. (C and D) Speed profiles for the same trials. (E and F) Variances in the same probe trials. The variance is reduced when subjects face a probe trial that is compatible with the structure of their previously experienced task.

To test specifically for the feedback component of structural learning, we had two groups make reaching movements to targets under two different visuomotor transformations that randomly changed at the start of each reach. One group experienced random rotations (rotation angles between −90° and +90°); the other group experienced random shearings (shearing parameters between −2.0 and +2.0; see Supplemental Experimental Procedures for details). Occasional probe trials that involved either 60° rotations or 1.5 shearings were introduced for both groups. Subjects from the two groups responded very differently for identical probe trials. For example, when presented with a rotation probe trial, the rotation group (red) and the shearing group (black) showed different hand paths (Figure 3A) and velocity profiles (Figure 3C). Generally, performance was faster when the probe-trial structure was compatible with the structure of the random trials (p < 0.001, paired t test). Moreover, the peak positional variance across probe trials showed a significant reduction (p < 0.005, one-tailed F test) in probe trials that were compatible with the learned structure, suggesting that exploration was reduced in these trials (Figures 3E and 3F). Although both groups might have adopted different control strategies, importantly, both tasks required the processing of feedback information in order to solve them. Therefore, feedback processing must be different in these two groups depending on previous experience. This suggests that the feedback control process is not generic but is highly dependent on the structure that subjects had experienced within a single trial, which argues against a nonspecific increase in feedback adaptability.

### Learning of 3D Rotations: Horizontal versus Vertical

**Figure 4:**
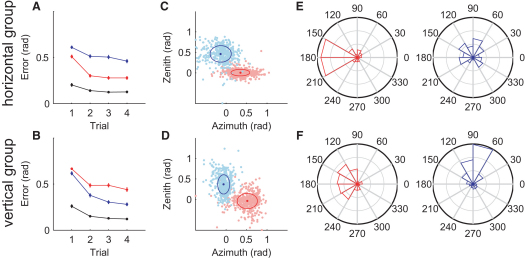
Structural Learning of 3D Rotations (A) Angular error in probe blocks of horizontal (red) and vertical (blue) 45° rotations experienced by a group that experienced random horizontal rotations before. There is a clear facilitation for learning the horizontal rotation. The black line indicates performance in the block of null-rotation (washout) trials preceding the probe block. (B) Performance error in the same probe blocks for a group that experienced random vertical rotations before. The facilitation pattern is reversed. (C and D) Movement variance shortly before trial end for both kinds of probe blocks. The variance in the task-irrelevant direction—perpendicular to the displacement direction—is significantly reduced for isostructural probe blocks (ellipses show the standard deviation). This suggests that subjects explored less outside the structure they had learned during the random rotation blocks. (E and F) Circular histograms of initial movement adaptation from the first trial of the probe block to the second trial. Subjects responded to probe blocks from the same structure in a consistent way, correcting toward the required target. In the case of probe trials for a different structure, subjects also showed components of learning in the direction of the previously learned structure.

To examine the final key feature of structural learning, structure-specific exploration, we had subjects make reaching movements to four targets in a three-dimensional (3D) virtual reality environment (Figure S1). This allowed us to create two orthogonal visuomotor transformation structures in which subjects experienced either random vertical rotations or random horizontal rotations (see Supplemental Experimental Procedures for details). The rotation parameter changed randomly every four reaches, covering the full range between −60 and +60°. We then probed learning by exposing subjects to four movements of null rotation (veridical feedback) to assess average learning and wash out any previous learning, followed by four movements with a visuomotor rotation of 45° either in the horizontal or the vertical direction. Movement error was assessed as the angle between the target and the cursor position at 9 cm into the movement. Both groups showed structure-specific facilitation (p < 0.01, Wilcoxon rank-sum test over average performance in all probe blocks). For example, there was a rapid facilitation when the horizontal rotation group experienced a horizontal rotation (Figure 4A, red) as opposed to a vertical rotation (blue). To examine the exploration strategy of subjects, we examined positional error at the end of the four reaching movements (Figures 4C and 4D). We found that the horizontal rotation group showed significantly smaller variance (p < 0.01, one-tailed F test on z deviations) in the vertical direction for probe trials of the same structure (Figure 4C, red) as compared to the performance of the vertical rotation group in the same trials (Figure 4D, red). Accordingly, we found a reverse pattern in the vertical rotation group (Figure 4D, blue compared to Figure 4C, blue; p < 0.01, F test on x deviation). Moreover, we found the same variance patterns in the feed-forward part of the movement (p < 0.01 in both cases; Figure S2). This suggests that subjects adjusted the variability of their movements so as to explore preferentially along the previously experienced structure.

To examine the direction of exploration, we examined the evolution of learning between the first and second probe trials (Figures 4E and 4F). For probe trials of the same structure, subjects showed rapid exploration along the structure (Figure 4E, red; Figure 4F, blue). However, for probe trials of the other structure, exploration also had components along the direction of the previously learned structure—the horizontal rotation group also showed horizontal components of exploration when exposed to vertical rotation probe trials (Figure 4E), whereas the vertical rotation group also showed vertical components of exploration when exposed to horizontal rotation probe trials (Figure 4F). The statistics of adaptation displayed in Figures 4E and 4F differ significantly (p < 0.05, two-sample Kolmogorov-Smirnov test for absolute angles of the red distributions and the blue distributions, respectively). Analysis of the performance in null-rotation (veridical feedback) trials that preceded the probe trials (Figures 4A and 4B, black line) shows that the exploratory behavior is not simply a consequence of incomplete washout because the errors in the unperturbed direction observed during the initial probe trials with unmatched structure exceed the corresponding errors in the preceding null-rotation trials (p < 0.005, two-sample t test). This means that subjects actively explored this direction and did not simply exhibit washout effects. Thus, both the group-specific exploration and the variance modulation in feed-forward as well as in feedback control support the concept of structure-specific exploration.

### Conclusion

Our results show that when subjects are exposed to randomly varying tasks of the same structure, the motor control process can extract the structure of the task and thereby exhibit structure-specific facilitation, interference reduction, and exploration. This suggests that the human motor system relies on structural learning for skill acquisition. Traditional models of sensorimotor learning have focused on function approximation during learning of sensorimotor mappings. Typically, learning the mapping from sensory inputs X to a motor command U has been conceptualized as a mapping $U=\sum_{i}w_{i}g_{i}\left(X\right)$, where $g_{i}\left(\cdot \right)$ are so-called basis functions and $w_{i}$ are the adjustable parameters of the mapping [2, 6, 15, 16]. Such a simple model of motor learning runs into problems in our case because of the presence of hidden variables that vary randomly over time (e.g., the varying rotation angle ϕ) —the model would simply learn the average mapping (e.g., the average over all angles). In our experiments, we deliberately set the expectation value of these hidden variables to zero (i.e., <ϕ>=0), so that if subjects were to simply learn the average of the experienced transformations [20–25], this would be the identity mapping—that is, no learning of the transformation would occur. Because subjects showed clear improvement after random training, they must have learned much more than the average mapping. They have learned how to adapt efficiently to related control tasks, and our results suggest that such facilitation is due to the common structure of the control tasks.

Processes of metalearning have been previously reported [26–28]. A recent study [27] has shown, for example, that learning rates in visuomotor adaptation depend on the error statistics. In particular, it was found that blurred visual feedback leads to slower adaptation rates, whereas high uncertainty in the visuomotor mapping leads to higher adaptation rates. However, this study did not examine how the structure of the mapping might influence adaptation. In our study, we found that uncertainty in the mapping can lead to both higher and lower adaptation rates (red and green curves in Figure 2), depending on the previously experienced task structure. In a similar vein, another recent study [28] has emphasized that facilitation of relearning a visuomotor mapping cannot be understood as a superposition of adaptation processes in the brain with constant adaptation rates, but rather that these rates should be adaptive as well. We also advocate such adaptability of adaptation rates, but the novelty in our study is that we show that these rate changes and the accompanying changes in movement variability and explorative behavior can be understood by structural learning both in the feed-forward and feedback component of movements. Understanding how feed-forward and feedback processes interact to achieve structural learning will be an interesting area of future research [29, 30].

The principle of structural learning is not confined to the motor system and could also govern processes of perceptual and cognitive learning [31, 32]. For example, cognitive “learning to learn” phenomena, in which subjects show facilitation in categorization or concept learning tasks if they have had random exposure to other items of a given category [33], can be recast as structural-learning phenomena. Structural learning might therefore provide a connection between motor learning and concept learning in cognitive neuroscience, given that scalable motor structures can be considered as a precursor to motor concepts [34]. Ultimately, understanding how recurrent neural networks [35] accomplish structural learning might elucidate the neural basis of the unsurpassed flexibility of biological controllers. In conclusion, we suggest a novel concept of facilitated learning for skill acquisition, in which general rules about a class of behaviors are extracted and used to facilitate adaptation, minimize interference, and guide exploration.

## Supplemental Data

Document S1. Supplemental Experimental Procedures, a Theoretical Perspective on Structural Learning, a Neurophysiological Perspective on Structural Learning, and Three Figures
