# Supplementary material for: Motor Task Variation Induces Structural Learning
Source: Curr Biol. 2009 Feb 24;19(4):352–7. doi: 10.1016/j.cub.2009.01.036 (PMC2669412; doi:10.1016/j.cub.2009.01.036)
Supplement: Document S1. Supplemental Experimental Procedures, a Theoretical Perspective on Structural Learning, a Neurophysiological Perspective on Structural Learning, and Three Figures [file mmc1.pdf]

Current Biology, Volume *19*

Supplemental Data

Motor Task Variation Induces Structural Learning

D.A. Braun, A. Aertsen, D.M. Wolpert, and C. Mehring

A. Supplemental Experimental Procedures
B. Structural Learning – A Theoretical Perspective
C. Structural Learning – A Neurophysiological Perspective
D. Supplemental References
E. Supplemental Figures

A. Supplemental Experimental Procedures

All experimental procedures were approved by the local ethics committee and subjects provided written informed consent. All subjects were students either at the University of Cambridge or at the University of Freiburg.

Experiment I. Subjects controlled a cursor on a computer screen by moving the end of a long (4m) pendulum that moved in the horizontal plane. Movements of the hand were tracked by an ultrasonic system (CMS20, Zebris Medical GmbH, Germany, 300 Hz sampling, 0.085 mm accuracy) and displayed to scale in real-time on the screen. The screen displayed 8 circular targets (radius 1.6 cm) arranged concentrically around a starting position (center-target distance 8 cm). Subjects were asked to move the cursor swiftly and in a straight line to each target. For a valid trial, the target had to be reached within 2s. Movement duration was defined as the time elapsed from the moment the center of the cursor crossed the circumference of the starting position to the moment the center of the cursor entered the target circle and remained within the target for at least 500ms. The structural learning group (26 subjects) was exposed to 800 random trials of visuomotor rotations about the starting position. The rotation transformation was:

$$x^{Cursor} = \begin{bmatrix} \cos(\phi) & \sin(\phi) \\ -\sin(\phi) & \cos(\phi) \end{bmatrix} \cdot x^{Hand}$$

The rotation angle changed every 8 trials and was drawn randomly from a uniform distribution between -90º and +90º. Within these 8 trials with a fixed rotation angle, each of the 8 targets was presented once in pseudo-random order. A naïve control group (18 subjects) performed an equal number of movements with veridical feedback. A third group (12 subjects) experienced random linear transformations that were composed of a combined rotation, shearing and scaling. In 50% of the trials an x-shearing was applied, and in the other 50% of the trials a y-shearing was applied such that either

$$x^{Cursor} = \begin{bmatrix} \cos(\phi) & \sin(\phi) \\ -\sin(\phi) & \cos(\phi) \end{bmatrix} \cdot \begin{bmatrix} 1 & k \\ 0 & 1 \end{bmatrix} \cdot \begin{bmatrix} s_x & 0 \\ 0 & s_y \end{bmatrix} \cdot x^{Hand}$$

or

$$x^{Cursor} = \begin{bmatrix} \cos(\phi) & \sin(\phi) \\ -\sin(\phi) & \cos(\phi) \end{bmatrix} \cdot \begin{bmatrix} 1 & 0 \\ k & 1 \end{bmatrix} \cdot \begin{bmatrix} s_x & 0 \\ 0 & s_y \end{bmatrix} \cdot x^{Hand}$$

The rotation angle was drawn from the uniform distribution between -90º and +90º, the shearing parameter was drawn between -5 and +5 and the scaling parameters were drawn between 0.3 and 3. Additionally, it was made sure that the combined transformation allowed movements within a 15cm workspace. Importantly, whenever the randomly selected rotation angle fell between +50º and +70º this group experienced a +60º rotation, and whenever the randomly selected rotation angle fell between -50º and -70º this group experienced a -60º rotations. Thus they experienced as many ±60º rotations as the random rotation group. After their different exposures all three groups then experienced a +60º rotation for 50 trials followed by a -60º rotation for 50 trials, followed by another +60º rotation for 50 trials. Performance was assessed as the integrated absolute deviation from a straight line to the target (cumulative error) and as the angular deviation (rad) at 200ms after movement onset (defined by a speed threshold at 3cm/s).

*Experiment II***.** Subjects held the handle of a vBOT robotic manipulandum that could be moved with minimal inertia in the horizontal plane [1]. The positions and velocities of the hands were calculated on-line at 1000 Hz. The hand position was displayed as a circular cursor (1 cm radius) in the plane of the arm by means of a rear-projection system. In every trial one of eight concentrically arranged targets (center-target distance 9 cm; target radius 2 cm) appeared and the movement to the target had to be executed within 1800 ms. The rotation group (4 subjects) experienced random rotations over 400 trials (as defined above). The rotation angle changed every trial and was drawn from a uniform distribution between -90º and +90º. The shearing group (4 subjects) experienced random horizontal shearings over 400 trials. The shearing transformation was:

$$x^{Cursor} = \begin{bmatrix} 1 & k \\ 0 & 1 \end{bmatrix} \cdot x^{Hand}$$

The shearing parameter k changed every trial and was drawn from a uniform distribution between -2.0 and +2.0. Both groups then experienced the same kind of random trials as before on 70% of trials. However, on 30% of the trials they experienced either a +60º-rotation probe trial or a horizontal 1.5-shearing probe trial. Shearing probe trials were limited to 4 of the 8 targets and the shearing had a positive sign for the upper targets and a negative sign for the lower targets — this allowed us to average the trajectories of these shearing probe trials as they were spatially identical relative to the line between start point and target. The trajectories were aligned with a speed threshold criterion at 10cm/s.

Experiment III. Subjects operated a 3 degree of freedom manipulandum (PHANTOM 1.5 Haptic Device, SensAble) with their index finger in a 3D virtual reality environment. A combination of stereoscopic monitor, mirror and crystal eye shuttered glasses allowed us to overlay 3D images onto the workspace of the manipulandum. Hand position and velocity was sampled at 1000 Hz. Four concentric target spheres (radius 1.25 cm) were projected on a plane orthogonal to the line of sight (depth

dimension) with an origin-target distance of 10 cm (see figure S1). The target had to be hit within 800 ms by a cursor (radius 1cm) representing the subjects finger tip. Movements were to be performed in mini-blocks of 4 trials to the four targets in a random order. Initially, the horizontal-rotation group (6 subjects) was exposed to 400 random rotations around the vertical-axis which corresponds to left-right displacements. The rotation angle changed over blocks of 4 trials and was drawn from a uniform distribution between -60º and +60º. The vertical-rotation group (6 subjects) was exposed to 400 random rotations around the horizontal-axis which corresponds to up-down displacements. The magnitude of the perturbation was the same for both groups. Both groups then continued to experience the same blocks of random rotations on 70% of the trials. However, on 30% of the trials there was a probe block of either 45º-horizontal rotations, or, equally likely, a probe block of 45º-vertical rotations. This probe block was always preceded by a block with veridical feedback, where a movement to each of the four targets had to be made. Performance in the probe blocks was assessed by the angle between the target and the cursor position at 9cm into the movement. End point spread was assessed as the difference in azimuth and zenith between the cursor position at 9cm movement distance and the target. To examine the error evolution we calculated the vector change in error in the xz-plane between the first and second probe trials at 9cm into the movement, and we plotted these changes in a circular histogram where the angles were computed from the vector difference in the xz-plane indicating the direction of adaptation. For the supplementary figure S2 we computed an initial angular error again at 200ms after movement onset (defined by a speed threshold at 3cm/s).

B. Structural Learning – A Theoretical Perspective

Structural learning in our experiments means that subjects have learned to extract the relationship between sensory inputs, hidden task variables and motor output. Formally, the relationship between the motor command *U*, the sensory input *X* and some internal variable μ that represents the hidden task variable can be expressed as $U = f(X, \mu)$. It should be noted that expressing the control command as $U = f(X, \mu)$ does not make any statement about the representation of *f*, and thus does not necessarily imply meaningful extrapolation, beyond the points of μ and X that were experienced during training. For instance, the motor command U could be represented by a mixture of local experts [2], such that $U = \sum_i \alpha_i(\mu) g_i(X)$, where $\alpha_i(\mu)$ is the weight of each expert g_i. In this case, the parameter μ defines a low-dimensional structure made up of local experts. In general, once a structure $f(X, \mu)$ is learned, adaptation and generalization can be conceived of as adapting a meta-parameter μ that shapes the control command according to the learned structure. Several previous findings can be cast within such a structural learning framework. For example, muscle synergies [3,4], generalized motor programs [5] and possibly even motor equivalence [6] can be regarded as structures that are scaled by “activation levels”.

In adaptive control theory $U = f(X(t), \mu(t), t)$ is called a *parametric* adaptive control law, because it encapsulates the knowledge of how the unknown parameters $\mu(t)$ are structurally related to the other control variables, such that estimating the parameters $\mu(t)$ amounts to solving a parametric optimization problem. Conversely, when there is

no explicit parameterization available that is suitable for a particular environment, the adaptive control problem is substantially more complex: the dimensionality of the control problem has to be established, the relevant control variables have to be extracted as well as their interrelations, range of potential values (e.g. discrete vs. continuous, etc.), time scales, stochasticity (e.g. noise levels, covariance, etc.) and other features. These issues constitute the realm of *structural* adaptive control [7,8]. With regard to our experiments structural learning would correspond to identifying a useful parameterization of the hand-cursor control (e.g. a rotation parameter for a visuomotor rotation) and establishing an adaptive control law to cope with the changing environment. It would be of great interest to investigate such adaptive control laws within the framework of adaptive optimal feedback control, given that non-adaptive optimal feedback control has successfully accounted for numerous motor behaviors in the recent past [9-12].

Adaptive optimal control models might also enhance our understanding of how feedforward and feedback components of structural learning are integrated in the nervous system. In our experiments we examined the initial component of movements (feedforward) as well as later stages in the movement (feedback) separately and this indicated that structural learning is evident in both the feedforward and the feedback pathway. An optimal feedback controller would use an adaptively scaleable internal model $M(\mu)$ in both feedforward and feedback control for adaptive sensorimotor integration. In a forward pathway an optimal feedback controller would use Bayesian estimation to obtain an optimal estimate of the state of the environment.(e.g. Kalman filter). In the beginning of the movement before feedback is available this estimate is mainly based on an internal model prior $M(\mu)$ determining feedforward control. In a feedback pathway an optimal feedback controller would estimate $\mu(t)$ online during the movement and adjust the feedback control law accordingly. Thus, if the motor system behaves like an adaptive optimal feedback controller then it would rely on adaptive internal models both in the feedforward and the feedback phase of the movement.

From a Bayesian point of view, structural learning implies that the learner must maintain a probability distribution over possible structures that could explain the data. Such structural learning is typically studied in the framework of Bayesian Networks (Fig. S3). A Bayesian Network is a graphical method to efficiently represent the joint distribution of a set of random variables [13-17]. In the case of sensorimotor learning these random variables could be N variables for the receptor input $R_1, R_2, ..., R_N$ (e.g. retinal input, proprioceptive input, or later stages of neural processing) and M variables for the motor output $U_1, U_2, ..., U_M$ (e.g. muscle activations or earlier stages of neural processing) (Fig. S3A). The dependencies between these variables are expressed by arrows in the network indicating the relation between any variable X_i (such as R_j or U_k) and its direct causal antecedents denoted as $parents(X_i)$. Thus, depending on a particular network structure S with model parameters μ_S the joint probability distribution $P(\vec{X}) = P(X_1, X_2, ..., X_{N+M})$ can be split up into a product of conditional probabilities: $P(\vec{X} \mid S, \mu_S) = \prod_{i=1}^{N+M} P(X_i \mid parents(X_i), S, \mu_S)$. The structure S of the network determines the dependencies between the variables—that is the presence or absence of arrows—while the probabilities that specify the actual

dependencies quantitatively are parameters of that structure. Therefore, 'structural learning' refers to learning the topology of such a network, whereas 'parametric learning' means determining quantitatively the causal connections given by the structure. In particular, the problem of structural learning is severe in the presence of hidden variables, because the structure underlying the observations has to be inferred. This is the standard case in sensorimotor learning. For instance, in the case of the rotation experiments the hidden variable we are interested in is the rotation angle. If the nervous system can extract this hidden variable the joint probability distribution over the sensorimotor space can be efficiently computed as $P(\vec{R},\vec{U}) = P(\vec{U} \mid \mu)P(\mu \mid \vec{R})$, where μ represents a rotation-specific hidden variable (Fig. S3B). Formally, the inference process during structural learning is split up into two steps: (a) computing the posterior probability $P(S \mid X)$ of a certain structural model *S* given the data *X*, and (b) computing the posterior probability $P(\mu_S \mid S, X)$ of the parameter μ_S given the structural model *S* and the data *X*. By using this formalism the concept of structural learning can be easily incorporated within the framework of Bayesian sensorimotor integration [18,19]. What is not shown explicitly in Fig. S3A,B is the time-dependence of the random variables $\vec{R}$ and $\vec{U}$. However, time can be easily included by extending the graph to a Bayesian Network that represents sequences of these random variables. This is called a Dynamic Bayesian Network [20] – compare Fig. S3C. The time dependence is vital for recognizing structural relationships between sensorimotor variables by means of motor task variation.

C. Structural Learning – A Neurophysiological Perspective

How could we imagine the process of structural learning in neurophysiological terms? As suggested in the introduction, we can imagine the brain as a controller with certain dials or '*free variables*', for example the synaptic configurations in the motor cortex. These free variables fluctuate and can be adjusted to lie on a manifold suitable to solve a given task [21]. Of course, such a manifold will in general have many dimensions due to the redundancy arising in the space of synaptic configurations, redundancy in effector kinematics and dynamics, and possibly task redundancy. Learning a particular task in this framework can generally be regarded as '*constraining'* the fluctuations of the weights in the space of synaptic configurations to a particular manifold. Thus, when several tasks have to be learned by the same neural circuitry, synaptic weights have to be adjusted to a configuration in the intersection of the manifolds optimal for these tasks [21]. Interestingly, if we assume synaptic noise in the motor system, learning more demanding tasks leads to more stable neural representations, because "when more task constraints are added the dimension of the manifold reduces, thus reducing the drift in synaptic strengths" [21].

Within this picture, structural learning would mean 'constraining' synaptic configurations to a manifold, which we can conceptualize as dimensionality reduction (Fig. 1). Accordingly, the on-line estimation process of the hidden variables μ can make use of these "tracks" laid down in the structural learning process. Thus, the process of on-line adaptation in our experiments is shaped by the structure that has

been learned before, which is why we observe different adaptation patterns – i.e. trajectories and variability patterns – depending on the experienced structure (e.g. Fig. 3). Importantly, the random task design imposes much more constraints on the control process than the traditional block design, because the motor system is forced to come up with a 'common explanation' for a plethora of input/output relationships on a short time scale. Taking into account synaptic noise as modelled in [21] this can explain why random exposure leads to better retention (see [22] for similar results in motor sequence learning) and why visuomotor learning in the traditional block design is much more susceptible to interference [23-26]: The reason is that many manifolds in the space of synaptic configurations have to intersect, which leads to a much lower-dimensional manifold and a more stable representation [21].

D. Supplemental References

1. Bays PM, Wolpert DM (2006) Actions and consequences in bimanual interaction are represented in different coordinate systems. J Neurosci 26: 7121-7126
2. Haruno M., Wolpert D, Kawato M (2001) MOSAIC model for sensorimotor learning and control. Neural Comp. 13:2201-2220
3. d'Avella A, Saltiel P, Bizzi E (2003) Combinations of muscle synergies in the construction of natural motor behavior. Nat Neurosci 6: 300-308
4. Tresch MC, Saltiel P, Bizzi E (1999) The construction of movement by the spinal cord. Nat Neurosci 2: 162-167
5. Schmidt RA (1975) A schema theory of discrete motor skill learning. Psychol Rev 82: 225-260
6. Lashley KS (1930) Basic neural mechanisms in behaviour. Psychol Rev 37: 1-24
7. Sastry S, Bodson M *Adaptive Control: Stability, Convergence, and Robustness.* (Prentice-Hall, 1994)
8. Barto AG *Connectionist learning for control.* In: Neural Networks for Control, Miller, W. T., Sutton, R. S. & Werbos, P. J. (eds.), (Cambridge, MA: MIT Press, 1996)
9. Todorov E (2005) Stochastic optimal control and estimation methods adapted to the noise characteristics of the sensorimotor system. Neural Comp 17: 1084-1108
10. Todorov E (2004) Optimality principles in sensorimotor control. Nat Neurosci 7: 907-915
11. Scott SH (2004) Optimal feedback control and the neural basis of volitional motor control. Nat Rev Neurosci 5: 534-546
12. Todorov E., Jordan MI (2002) Optimal feedback control as a theory of motor coordination. Nat Neurosci 5: 1226-1235
13. Pearl J, Russell S *Bayesian Networks*, in M. A. Arbib (Ed.), Handbook of Brain Theory and Neural Networks, pp. 157–160, (Cambridge, MA: MIT Press, 2003)
14. Jensen FV *Bayesian Networks and Decision Graphs.* (Springer Verlag, 2001)
15. Heckerman D (1995) A Tutorial on Learning With Bayesian Networks, Technical Report MSR-TR-95-06 (see ftp://ftp.research.microsoft.com/pub/tr/tr-95-06.pdf)
16. Needham CJ, Bradford JR, Bulpitt AJ, Westhead DR (2007) A Primer on Learning in Bayesian Networks for Computational Biology. PLoS Comp. Biol. 3: e129
17. Körding KP, Wolpert DM (2004) Bayesian integration in sensorimotor learning, Nature 427: 244-247
18. Körding KP, Wolpert DM (2006) Bayesian decision theory in sensorimotor control, Trends Cogn Sci 10: 319-326
19. Doya K, Ishii S, Pouget A, Rao RPN *Bayesian Brain. Probabilistic Approaches to Neural Coding.* (Cambridge, MA: MIT Press, 2003)
20. Ghahramani Z (1997) Learning Dynamic Bayesian Networks. Lecture Notes in Computer Science 1387: 168-197
21. Rokni U, Richardson AG, Bizzi E, Seung S (2007) Motor learning with unstable neural representations. Neuron 54:653-666
22. Shea JB, Morgan RL (1979) Contextual interference effects on the acquisition, retention and transfer of a motor skill. J. Exp. Psychol. 5: 179-187

23. Krakauer JW, Ghilardi MF, Ghez C (1999) Independent learning of internal models for kinematic and dynamic control of reaching. Nat. Neurosci. 2: 1026-1031
24. Tong C, Wolpert DM, Flanagan JR (2002) Kinematics and dynamics are not represented independently in motor working memory: evidence from an interference study. J. Neurosci. 22: 1108-1113
25. Bock O, Schneider S, Bloomberg J (2001) Conditions for interference versus facilitation during sequential sensorimotor adaptation. Exp Brain Res 138: 359-365
26. Wigmore V, Tong C, Flanagan JR (2002) Visuomotor rotations of varying size and direction compete for a single internal model in motor working memory. J Exp Psychol Hum Percept Perform 28: 447-457

D. Supplemental Figures

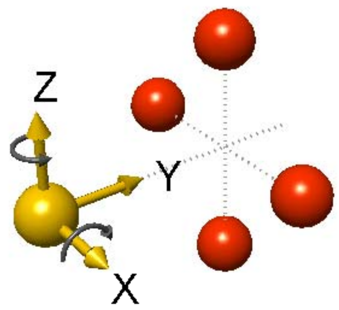

Figure S1. Schematic of experimental setup. Four targets (red spheres) were arranged concentrically in a plane perpendicular to the line of sight (y-dimension). The starting position (green sphere) was 10 cm away from each target. Rotations were either horizontal (i.e. rotation around z-axis) or vertical (i.e. rotation around x-axis).

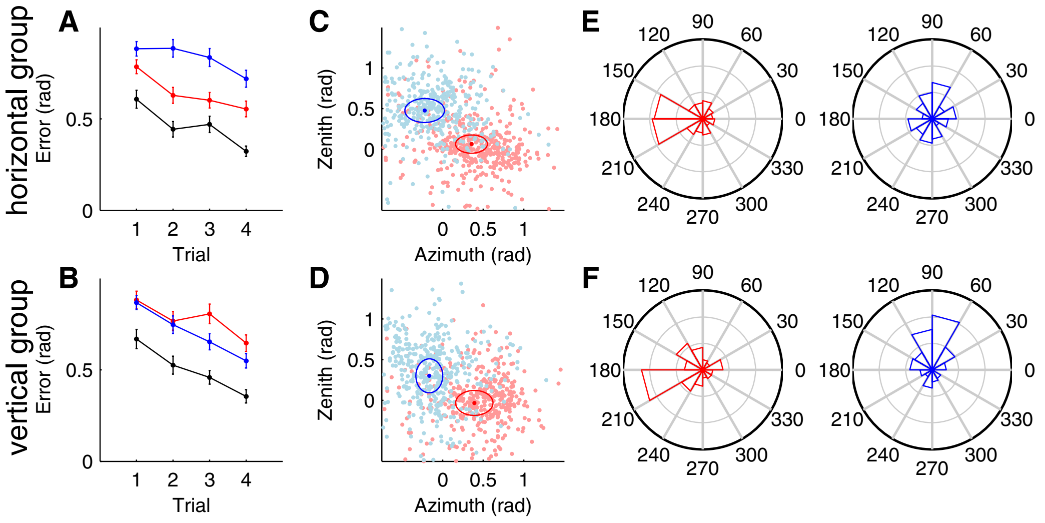

Figure S2. Structural learning of 3D rotations (initial component at 200ms into the movement). *(A)* Error in probe blocks of horizontal (red) and vertical (blue) 45º-rotations experienced by a group that experienced random horizontal rotations before. There is a clear facilitation for learning the horizontal rotation. The black line indicates performance in the preceding block of standard trials. *(B)* Performance error in the same probe blocks for a group that experienced random vertical rotations before. The facilitation pattern is reversed. *(C,D)* Initial movement variance for both kinds of probe blocks. The variance in the task-irrelevant direction—perpendicular to the displacement direction—is significantly reduced for isostructural probe blocks (ellipses show variances). This suggests that subjects explored less outside the structure they had learned during the random rotation blocks. *(E,F)* Circular histograms of initial movement adaptation from the 1st trial of the probe block to the 2nd trial. Subjects responded to probe blocks from the same structure in a consistent way correcting towards the required target. In case of probe trials for a different structure, subjects showed a tendency for less consistent responses.

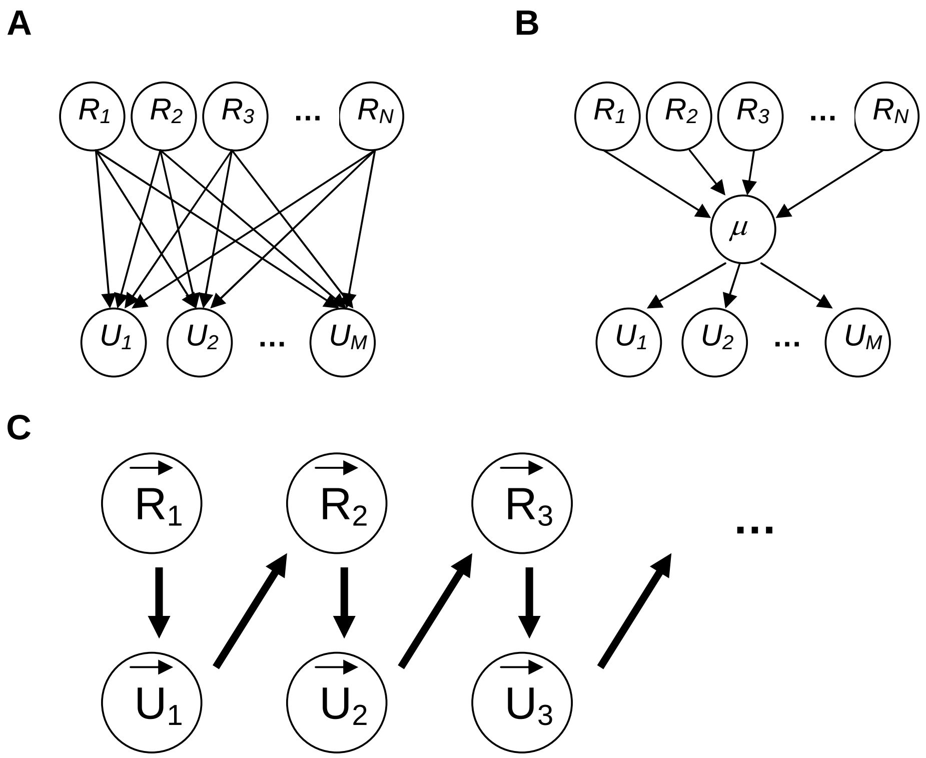

Figure S3. Structural learning in Bayesian Networks. *(A)* The nodes of the Bayesian Network represent random variables such as sensory inputs R_j and motor outputs U_k. The arrows indicate causal dependencies that are usually expressed via parameterized probability density functions. Learning the parameters of the full joint probability distribution in this network will require substantial computations. *(B)* In this network there is a hidden variable μ, that corresponds to what we have called a 'meta-parameter'. The joint probability distribution over all variables splits up into a product of conditional distributions with regard to μ. This substantially reduces the dimensionality of the parameter space. In our experiments μ corresponds for instance to internal variables specific for rotations. (C) A temporal Bayesian network where the arrows on top of the variables indicate vector notation and the lower case indices indicate time steps.
